# Supplementary material for: MALDI-TOF: A new tool for the identification of Schistosoma cercariae and detection of hybrids
Source: PLoS Negl Trop Dis. 2023 Mar 28;17(3):e0010577. doi: 10.1371/journal.pntd.0010577 (PMC10081743; doi:10.1371/journal.pntd.0010577)
Supplement: S1 Table — (DOCX) [file pntd.0010577.s001.docx]

| **Spectra name** | **Species** | **Date of cercaria emission** |
| --- | --- | --- |
| LFDC41 | *Alaria alata* | 12/06/2017 |
| LFDC53 DD | *Alaria alata* | 12/06/2017 |
| LFDC54 ED | *Alaria alata* | 12/06/2017 |
| LFDC43 DD | *Cotylurus sp.* | 12/06/2017 |
| LFDC42 | *Australapatemon sp.* | 12/06/2017 |
| LFJO2 | *Australapatemon sp.* | 12/06/2017 |
| 1SHC 28012022 | Corsican hybrid | 10/07/2020 |
| SHC 28012022 | Corsican hybrid | 10/07/2020 |
| LFDR1 | *Diplostomum pseudospathaceum* | 26/10/2017 |
| LFDR3 | *Diplostomum pseudospathaceum* | 26/10/2017 |
| LFDR4 | *Diplostomum pseudospathaceum* | 26/10/2017 |
| ECDC16 | *Echinoparyphium sp.* |  |
| ECJO3 | *Echinostoma revolutum* | 09/08/2017 |
| 1F1 28012022 | F1 hybrid : ♀ *S. haematobium x* ♂ *S. bovis* | 10/07/2020 |
| F1 28022021 | F1 hybrid : ♀ *S. haematobium x* ♂ *S. bovis* | 10/07/2020 |
| 2F1-prime 28012022 | F1’ hybrid: ♂ *S. haematobium x* ♀ *S. bovis* | 10/07/2020 |
| 2F-prime1 28012022 | F1’ hybrid: ♂ *S. haematobium x* ♀ *S. bovis* | 10/07/2020 |
| 5F-prime1 28022021 | F1’ hybrid: ♂ *S. haematobium x* ♀ *S. bovis* | 10/07/2020 |
| EDCR1 ED centri | *Petasiger phalacrocoracis* | 26/10/2017 |
| EDCR2 | *Petasiger phalacrocoracis* | 26/10/2017 |
| X1DR2 | *Plagiorchis sp* | 26/10/2017 |
| XIDR3 | *Plagiorchis sp* | 26/10/2017 |
| SB2 28012022 | *Schistosoma bovis* | 06/11/2019 |
| SB3 22082021 | *Schistosoma bovis* | 06/11/2019 |
| SB4 28082022 | *Schistosoma bovis* | 06/11/2019 |
| SH 28012022 | *Schistosoma haematobium* | 06/11/2019 |
| SHP1 28012022 | *Schistosoma haematobium* | 10/07/2020 |
| SHP2 28012022 | *Schistosoma haematobium* | 10/07/2020 |
| MS1 28012022 | *Schistosoma mansoni* | 06/11/2019 |
| MS2 28012022 | *Schistosoma mansoni* | 06/11/2019 |
| MS3 28012022 | *Schistosoma mansoni* | 06/11/2019 |
| SR1 28012022 | *Schistosoma rodhaini* | 06/11/2019 |
| SR2 28012022 | *Schistosoma rodhaini* | 06/11/2019 |
| SR3 28012022 | *Schistosoma rodhaini* | 06/11/2019 |
| FODR1 | *Trichobilharzia anseri* | 26/10/2017 |
| LFZE1 | *Tylodelphys sp.* | 25/11/2017 |
| LFZE2 | *Tylodelphys sp.* | 25/11/2017 |
| LFZE3 | *Tylodelphys sp.* | 25/11/2017 |

Table S1 MSP database composition
