# Supplementary material for: MALDI-TOF: A new tool for the identification of Schistosoma cercariae and detection of hybrids
Source: PLoS Negl Trop Dis. 2023 Mar 28;17(3):e0010577. doi: 10.1371/journal.pntd.0010577 (PMC10081743; doi:10.1371/journal.pntd.0010577)
Supplement: S1 Fig — (PDF) [file pntd.0010577.s002.pdf]

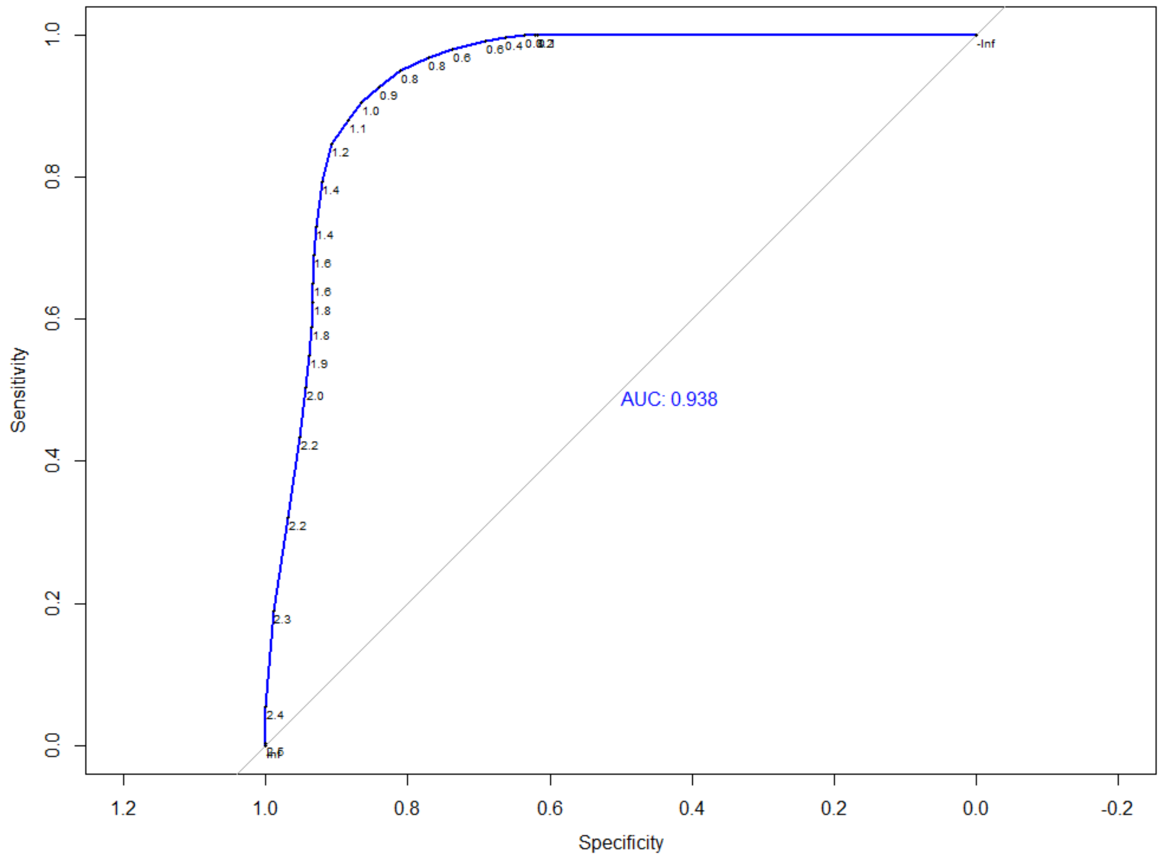

Figure S1 ROC plot of LSV Value for specific identification of cercariae in the blind-test dataset.

Best threshold according to Youden method is 1.05 with specificity and sensitivity of respectively: 0.8652424 and 0.9053398.
