# Supplementary material for: MALDI-TOF: A new tool for the identification of Schistosoma cercariae and detection of hybrids
Source: PLoS Negl Trop Dis. 2023 Mar 28;17(3):e0010577. doi: 10.1371/journal.pntd.0010577 (PMC10081743; doi:10.1371/journal.pntd.0010577)
Supplement: Attachment — Submitted filename: Reviewer response-14122022.docx [file pntd.0010577.s004.docx]

Dear editor,

Reviewer's Responses to Questions


**Reviewer #1: The result showed the big difference between the different species of parasite in Fig 1. Some peaks above m/z 10000 in *Schistosoma haematobium* and *bovis*, Does the author check that and get the peptide sequence?**

Obtaining peptide sequences with MALDI-TOF MS is more difficult than with ESI-TOF MS/MS due to the non-fragmentation of ions. The usual solution to this issue, is to isolate the proteins, for example by electrophoresis in 2 dimensions, then to apply a tryptic digestion and to analyze the spectrum obtained with a database containing proteins digested in-silico such as Mascot.

In our approach we perform whole cell analysis without prior protein isolation which makes a trypsin approach too difficult.

One possibility is to calculate the m/Z of each protein in the genome of the organism of interest and look for a peak corresponding to this m/Z (taking into account that protein ionized usually in mono or di-charged ions). This has been done with ribosomal proteins to provide biomarkers for bacterial identification in the RiboPeaks database (Tomachewski Bioinformatics 2018).

In the case of high molecular weight proteins, this approach is quickly limited by the mass error inherent to the technic which makes several proteins correspond to the same peak. The one peak corresponds to one protein approach is valid only on condition that the peaks considered correspond to whole proteins which ionize well in an acidic environment. In addition, during ionization, the hydrogen bonds and even the disulphide bridges which maintain the various protein chains can break.

Moreover, the automatic translation of the *Schistosoma* genomes into putative proteins does not take into account post-transcriptional or translational modifications.

For example, we predicted (using R Peptides package) from protein sequences of *S. haematobium* and *S. bovis* found on uniprot their m/Z for monocharged and discharged ions (mz1 and mz2), the mass error (300ppm considering the calibration of the device) and ranges it in which the peaks for ions must be (included in the interval between me_low_mz/me_high_mz). The data could be downloaded here: <https://ssds-cloud.univ-reims.fr/index.php/s/L45FDYH22Cfeayn> (excel file named ProtHMW Schistomz-14122022.xlsx in "Uniprot Shematobium_fasta_query" tabs).

The values of m/Z >10000 of a representative spectra of *S. haematobium* were extracted (Flexanalysis software bruker daltonics). They very frequently correspond to several of the proteins present on Uniprot. The first four hits of each peak (mono-charged ions only) are reported in the "SH1B_0_A12_1_masslist" tabs.

It is therefore not possible for us to find the amino acid sequence corresponding each MALDI-TOF peak and to compare them between the different species.

This very interesting question could be investigated by using another mass spectrometry technique such as ESI-TOF MS/MS and this could be an interesting clue for further studies.

**All the worm parasites were collected from the host in laboratory, could you please evaluate the detection mode with the wide type of cercaria from the field?**

We currently don’t have wild-type specimens from the field available.

Our previous study on MALDI-TOF identification of cercariae (Huguenin et al. Parasite 2019 PMID: 30838972) was based on field’s specimens. In this paper we demonstrate that MALDI-TOF is able to identify Trematoda’s species regardless of the host or the geographic origin. For example a perfect congruence was obtained between spectra of the same Trematode species from different snails from the same site (Ex: *Alaria alata* on *Anisus sp*. and on *Planorbis planorbis* in the Chambord site; *Australapatemon* on *Planorbis planorbis* and *Stagnicola* at Chambord), but also of the same species of snails from two sites distant from more than 100 km (*Australapatemon* on *Stagnicola*).

Moreover, we propose in this article to validate the diagnostic accuracy of MALDI-TOF using parasites that have been genetically very well characterized (i.e. whole genome sequencing). This aspect is particularly relevant in a context of introgressive hybridization. Genetic introgression is a continuous process and typing cercariae by maldi-tof supposes in parallel a complete genomic study of the same cercarial clonal population. This huge work could be the subject of future studies as we proposed in the discussion (line 420-426)

An evaluation in the field also raises interesting questions about the logistical organization and the medium for preservation of the specimens until analysis by MALDI-TOF. These points deserve to be dealt with in a new paper evaluating the performance of this technique in a real situation for a malacological investigation.

**The author described that “Molecular biology techniques allow a reliable diagnosis but are expensive. TOF is a recent technique that permits an inexpensive”, but I think the MALDI-TOF is not inexpensive but high throughput, so it maybe not expensive if you can solve the problem of sample size.**

Indeed, MALDI-TOF Mass Spectrometry requires a significant investment (~50-100 K€) to purchase the instrument and ensure its maintenance. However, the consumable cost is very low (especially for the Bruker system with washable targets). The cost drops very quickly in the case of high-throughput identification: Ten years ago the cost was evaluated to 0.41$ per bacterial identification for the clinical microbiology laboratory of a 3,046-bed university hospital (Gaillot et al. 2011 JCM PMID: 21998417). In 2015, Tran et al. claimed that the use of MALDI-TOF MS equated to 87.8% of reagents costs compared to conventional technics, the purchase cost of the equipment being amortized after 3 years (Tran et al. JCM 2015 PMID: 25994167). Therefore, in developed country MALDI-TOF has become the routine technic for identification of bacteria and yeasts. However endemic country for schistosomiasis may have an access problem to MALDI-TOF equipment especially for small and medium sized laboratories. This issue is discussed further in response to reviewer 2.

**The study of this paper is interesting and seems promising, the accuracy of the learning machine is good for detection, however, it could not detect the snail, which is important for the policy of schistosomiasis control and it may waste too much time for the parasite grown to the stage of cercaria.**

Hamlili et al. have recently published an interesting paper (Hamlili et al. 2020 Plos NTD PMID: 34516582), demonstrating the performance of MALDI-TOF for the specific identification of aquatic snails, intermediate hosts of Schistosoma’s. They propose an easy protocol for obtaining good quality spectra from the foot of frozen specimens and their spectral database (representing nine species) could be freely downloaded on: <https://www.mediterranee-infection.com/wp-content/uploads/2021/08/raw-data-frozen-and-ethanol-stored-snails.zip>. In a malacological study, MALDI-TOF could then be used both by anyone with access to MALDI-TOF for specific identification of emitted cercariae (with our database shared on Zenodo under the doi 10.5281/zenodo.6631761) and of emitting or non-emitting snails (with the database shared by Hamlili et al.). We try to clarify this issue in discussion lines 498-500.

**MALDI-TOF technique has been applied in the early detection of schistosomiasis, some reference should be noticed with PMID 31166908.**

The team of the Jiangsu Institute of Parasitic Diseases have indeed published several papers on the use of MALDI-TOF for detecting biomarkers of *S. japonicum* first in a rabbit model ( 2011 Int. Journal of Mass Spectrom. DOI: 10.1016/j.ijms.2010.10.013) then in mice used as sentinel (2016 Parasitol. Res. PMID: 27469535) and finally in sera of patients with newly developed advanced schistosomiasis (2018, Parasite PMID: 31166908). We first thought that these works were off-topic (studies on biomarkers in sera) in relation to our problem (specific identification of cercariae). Finally, we agree with reviewer 1 that this is an important work in this field of research. We added a sentence to mention these works in the introduction (line 118-121).

**Some minor error: Spell error for the word of schistosomiasis in Author Summary.**

It has been corrected, thank you for pointing.

**Reviewer #2: This is an interesting manuscript that reports research on an innovative idea, i.e. the application of MALDI-TOF mass spectrometry, a common routine diagnostic method for identification of bacteria and fungi in the microbiology lab, for identification of cercariae stemming from Schistosoma. The authors found that a reliable identification was achieved for most different cercariae, with exception of hybrids between *S. haematobium* and *S. bovis*, which were ‘misclassified’ as *S. haematobium*. The identification could be further improved using artificial intelligence algorithms.**

**I have some observations that might be addressed by the authors:**

**1) Abstract: Please spell out MALDI-TOF at first mention**.

Thank you, it has been corrected.

**2) Introduction: Well written chapter. Line 100: The official name of Ivory Coast is Côte d’Ivoire (should not be translated), please correct.**

Thank you, it has been corrected. We also corrected “Senegal” as “Sénégal” but let “Cameroon” as English is one of the two official languages of this country.

**3) Introduction, line 109: Please define MALDI-TOF at first mention. Also, I would not describe it as a “new technique” for bacteria identification as it has been used for almost 15 years in this context.**

It has been corrected. Indeed, MALDI-TOF has reached maturity in routine clinical microbiology, it's time to stop our habit of calling it a new technique

**4) Methods: Accurate description of the work, well written. Lines 210-212: In bacteriology, log score values (LSVs) >1.7 are usually considered as genus-specific and LSVs >2.0 as species-specific. Even though it remains unclear whether this applies likewise to parasites, it would be useful/desirable to report identification rates for both established cut-offs in the Results section (and perhaps in the Abstract). This should also be mentioned more clearly in the Methods and/or Discussion, because in the current form, LSVs >1.7 are repeatedly described as species-specific, e.g. in the first sentence of the Discussion. This should be modified, as it might be misleading.**

In our previous work on Trematoda’s cercariae (Huguenin et al. Parasite 2019 PMID: 30838972) we establish a cut-off of 1.7 for species specific identification. We add a ROC-curve as a supplementary file supporting that the cut-off of 1.7 is correct for species identification and in the present situation could even be lowered. We agree that this issue need to be developed. This issue is explained in the Results line 275-277.

**5) Discussion, line 417: typo error, please correct to S. guineensis.**

It has been corrected

**6) Discussion, lines 423-430: The significant influence of ethanol on spectra quality is a little surprising, as it differs from previous papers on MALDI-TOF for parasites, e.g. PubMed ID 34683327. Please mention and discuss this issue.**

The influence of ethanol on the MALDI-TOF spectra varies according to the studies: It is likely that in arthropods, ethanol conservation alters spectra (discussed by Sevestre et al. Future Microbiol. 2021, based on Nebbak et al. Med. Vet. Entomol. 2017). Zan Diarra et al. have even proposed a “de-alcoholization” protocol for analysing ticks preserved in ethanol (Zan Diarra et al. Plos NTD 2017).

Hamlili et al. have observed that ethanol storage significantly modify spectra profiles and decrease the performance for identification despite the use of de-alcoholization protocol. The authors proposed the use of a distinct database for specimens preserved in ethanol.

Concerning helminths, only one paper by Wendel et al. has compared the different storage media for the identification of *Taenia saginata* (Wendel et al. microorganism 2021). The author demonstrate that high quality spectra could be obtain form proglottids stored at -20°C in LC-MS grade water, 70% ethanol and NaCl 0.9% during the 24 weeks of the study period (evaluated in terms of Log-Score values).

As the team who publish this paper has kindly accepted to share their raw spectra with us, we used our R scripts to compare the different storage media in terms of signal intensity and signal to noise ratio. We don’t observed significant degradation of both total intensity of peaks and signal to noise ratio with ethanol, supporting the author’s conclusions.

Figure 1 : Boxplot of total intensity of peaks according to conservation methods and delay, for *T. saginata* proglottids based on the work of Wendel et al.

Figure 2 Boxplot of signal/noise ratio of peaks according to conservation methods and delay, for *T. saginata* proglottids based on the work of Wendel et al.

But as emphasized by the authors of this interesting paper, their results are restricted to *T. saginata* proglottids, and it does not permit to conclude that ethanol is a good preservative for all life-stages of all helminths.

In our previous paper on MALDI-TOF analysis of cercariae (Huguenin et al. Parasite 2019 PMID: 30838972), we already observed spectral quality degradation related to ethanol fixation with an important effect on spectra intensity.

As ethanol preservation is cheap and suitable for fields studies we will in future studies seek ways to obtain better performance with that kind of specimens. Our leads were using a specific MALDI-TOF MS database as proposed by Hamlili et al. or using machine learning to found “robust peaks” permitting identification whatever the conservation method used.

This issue has been developed in discussion: lines 433-452.

**7) Discussion: In the limitations of the work, it should be mentioned that many small- and medium-sized laboratories in the tropics do not (yet) have access to MALDI-TOF machines.**

We agree with this reviewer remarks. Even large university hospital in Africa, do not have a MALDI-TOF spectrometer and send their strains to Europe for identification confirmation by MALDI-TOF in the context of epidemiological studies (Schmider J et al. Tropical Medicine and Infectious Disease 2022, Tufa TB et al. Antimicrobial Resistance & Infection Control. 2022, Ombelet S et al. BMC Infectious Diseases. 2022). MALDI-TOF seems to be more accessible in Asia, were a survey showed that 27/219 (12.3%) laboratory diagnosing fungal infections use MALDI-TOF (Chindaporm Med. Mycol. 2018 PMID: 29036605).

As the use of MALDI-TOF has developed over the past ten years, some devices can be second-hand purchased (A Bruker Microflex LT can be purchased for around 25,000 euros on Ebay: <https://www.ebay.fr/itm/304460168404?hash=item46e33d7cd4:g:bwEAAOSwKyxffGPd&amdata=enc%3AAQAHAAAAoFbuueLyMXXj%2BbjBfTL5xV9RMrKASdQq2uZ%2FIqeLD%2ByPCAXkERn%2FaxCnK7CL2oO5TjkcYcexBk4unDFXMayUvWY44Shuj29jwsQtzGxI%2Bl2tSC9OLcWadP5snBq6wIk2VzRx3mD69u8xHQGn4f7brpJA6DeTjP0mJZDb0hHjXkRVjzMm2fzKLnXowenNN8i8QHmkP%2FAMvxrCdSDnazP1Gc8%3D%7Ctkp%3ABk9SR_zoz5XhYA>). But MALDI-TOF mass spectrometers are fragile devices requiring regular maintenance, which is not accessible to laboratories in southern countries.

Some reports show the interest of MALDI-TOF for tropical disease laboratories (Bulane et al. Afr. J. Lab. Med. 2017). MALDI-TOF has been proven particularly used for diagnostic and epidemiological purpose in the “Senegalese Network For The Exploration Of Nonmalarial Causes Of Fever” (Sokhna et al CID 2017 PMID: 28859342).

In the immediate future, the use of MALDI-TOF for a field malacological investigation will pose logistical problems for the transport of specimens to a reference laboratory in the endemic country or abroad. In this context, a robust, inexpensive preservation technique that does not require refrigerated transport would be particularly useful.

This issue is developed line 506-514.

We thank all the reviewers for their constructive comments that have significantly improved this paper.
