# Supplementary material for: MALDI-TOF: A new tool for the identification of Schistosoma cercariae and detection of hybrids
Source: PLoS Negl Trop Dis. 2023 Mar 28;17(3):e0010577. doi: 10.1371/journal.pntd.0010577 (PMC10081743; doi:10.1371/journal.pntd.0010577)
Supplement: Attachment — Submitted filename: reviewer comments.docx [file pntd.0010577.s005.docx]

1. In the part of the introduction, I think the cited epidemiological data on schistosomiasis are too antiquated and suggest searching for the availability of the latest disease data. (lines 62-66)
2. There may be some errors in the references in the Introduction section, it is recommended that the author checks for modification. (lines 117-119)
